# 硼替佐米治疗多发性骨髓瘤诱发MAS样病变并引起SIADH 3例报告并文献复习

**DOI:** 10.3760/cma.j.issn.0253-2727.2022.01.015

**Published:** 2022-01

**Authors:** 丽丽 王, 传营 耿, 艳茹 章, 垠 吴

**Affiliations:** 首都医科大学附属北京朝阳医院血液科，北京 100020 Department of Hematology, Beijing Chao-yang Hospital, Capital Medical University, Beijing 100020, China

蛋白酶体抑制剂硼替佐米是多发性骨髓瘤（MM）的一线治疗药物。文献报道，硼替佐米诱发低钠血症的发生率为15％～40％，其原因尚不明确[1]。本文报道了本中心3例MM患者应用含硼替佐米方案化疗后出现低钠血症伴高热、IL-1β和IL-6炎症因子升高、铁蛋白显著升高及多系统损害。这些表现不完全符合巨噬细胞活化综合征（macrophage activation syndrome，MAS）的典型表现，我们称其为“MAS样病变”。1例患者应用依托泊苷联合地塞米松治疗后临床症状缓解。本文旨在提示应用硼替佐米化疗期间应严密监测血清钠水平，持续发热的患者需警惕是否诱发了MAS样病变及抗利尿激素分泌不当综合征（syndrome of inappropriate antidiuretic hormone secretion，SIADH）。

## 病例资料

例1，男，50岁，2018年4月25日明确诊断为：MM，IgG-λ型，DS分期ⅢB期，ISS分期Ⅲ期，2018年5月1日开始接受第1个疗程PDD（硼替佐米+脂质体阿霉素+地塞米松）方案化疗。2018年5月22日行第2个疗程PD（硼替佐米+地塞米松）方案化疗。两次均于化疗第4天出现发热、咳嗽，抗感染治疗效果不佳。第2次化疗中肺CT示双下肺大片实变影，双侧胸腔积液，左下肺膨胀不全，考虑为“机化性肺炎”。予甲泼尼龙40 mg每日1次静脉滴注抗炎治疗2周后体温逐渐降至正常，复查胸部CT较前明显吸收。2018年6月27日开始第3个疗程PD方案化疗。化疗第4天患者开始出现体温升高，波动在39～40 °C。患者出现精神减弱，血压降至90/60 mmHg（1 mmHg＝0.133 kPa）左右，心率升至130～140次/分。实验室检查：PLT 50×10^9^/L，纤维蛋白原（FBG）570 mg/L，纤维蛋白原降解产物（FDP）320 mg/L；血清铁蛋白3470 µg/L；血淀粉酶532 U/L（正常参考值35～135 U/L），尿胰蛋白酶原Ⅱ阳性。血钠进行性下降至最低110.8 mmol/L，血渗透压224 mmol/L，尿钠475 mmol/24 h。胸部CT无明显异常；腹部CT示胰头肿胀，周围有渗出。考虑患者存在免疫性胰腺炎、凝血系统损伤、神经系统损伤等及SIADH。

结合既往3个周期硼替佐米联合化疗后相关表现，考虑可能为硼替佐米诱发了MAS样病变及SIADH，但未行细胞因子及噬血细胞性淋巴组织细胞增生症（HLH）相关检测。予甲泼尼龙抗炎，托伐普坦纠正低钠血症，中药退热，同时予禁食水、补液、生长抑素等对症治疗后患者精神好转，体温逐渐降至正常，血钠及血尿淀粉酶正常。后续调整为Rd（来那度胺+地塞米松）方案化疗，未再发生上述反应。

例2，男，54岁，2019年4月明确诊断为：MM，IgG κ型，DS分期ⅠA期，ISS分期Ⅱ期。2019年4月16日开始接受VRD（硼替佐米+来那度胺+地塞米松）方案化疗，5个疗程后评估疗效为VGPR（非常好的部分缓解），既往化疗过程顺利。2019年8月31日开始第6个疗程VRD方案化疗，化疗第2天饮冷水后出现腹泻伴发热，体温持续升高至40.3 °C，呈弛张热。腹泻每日3次，为水样便，便常规无异常，蒙脱石散对症治疗3 d后好转。呕吐3次，为胃内容物，呈喷射状。逐渐出现精神减弱，反应迟钝。查体未见阳性体征，脑膜刺激征阴性，病理反射阴性。实验室检查：2019年9月6日血钠最低降至114.8 mmol/L，血尿素氮（BUN）2.84 mmol/L，血渗透压241 mmol/L；4 h尿钠48.3 mmol；甲状腺功能：高敏促甲状腺激素（sTSH）0.05 µIU/ml；皮质醇节律：70 ng/ml（0:00），264 ng/ml（8:00），130 ng/ml（16:00）。脑脊液压力11 cmH_2_O（1 cmH_2_O＝0.098 kPa），微量总蛋白0.80 g/L（正常参考值0.15～0.45 g/L）。血EB病毒DNA 7.63×10^5^ IU/L，余细菌、真菌、病毒、支原体、衣原体、军团菌等病原学检查及便常规均未见阳性。脑电图、胸部CT、腹部CT、肾上腺CT、鞍区增强MRI、腹部超声、超声心动图均未见明显异常。

先后予患者多种抗细菌、抗真菌、抗病毒药物，体温无明显下降。静脉联合口服（约27 g/d）补钠无效，血钠仍进行性降低。内分泌科会诊意见：患者皮肤颜色正常，无色素沉着，无毛发脱落，鞍区增强MRI示脑垂体未见异常，肾上腺CT、甲状腺功能未见明显异常，无肝硬化、肾衰竭和心力衰竭等疾病。入院后血压波动在140～150/75～88 mmHg，无血容量不足依据。化验血Na^+^<135 mmol/L，血浆渗透压<275 mmol/L，在未使用利尿剂或未严格限制钠摄入的情况下尿Na^+^>40 mmol/L，血尿酸（UA）<200 µmol/L，BUN<4.5 mmol/L，肌酐（Cr）<80 µmol/L，生理盐水纠正低钠血症无效，考虑诊断为SIADH。SIADH不会引起发热，发热与SIADH无关。予患者限水（每日饮水量约为1000 ml），口服托伐普坦3.75 mg第1天、7.5 mg第2天、15 mg第3～10天，复查血钠升至131.6 mmol/L，体温逐渐降低至38.0 °C左右，精神较前好转。

患者加用托伐普坦后体温波动于37.8～38.0 °C，伴轻度低钠血症，托伐普坦15 mg每日1次。2019年9月细胞因子检测：IL-1β 54.6 pg/ml（正常参考值0～5.0 pg/ml），IL-6 7.36 pg/ml（正常参考值0～5.9 pg/ml），IL-8 895 pg/ml（正常参考值0～62 pg/ml），IL-10<5.00 pg/ml（正常参考值0～9.10 pg/ml），TNF-α 8.44 pg/ml（正常参考值0～8.10 pg/ml）。考虑患者持续高热、严重低钠血症可能为体内炎症因子风暴所致。予IL-6受体单克隆抗体托珠单抗注射液160 mg静脉滴注后约7 d血钠逐渐恢复至正常，托伐普坦逐渐减量并停药。但在用药3 d后患者体温峰值再次较前升高，至用药后第9天体温最高可升至41.2 °C，且逐渐出现嗜睡、不能正确回答问题等神志改变。逐渐出现心肌肌钙蛋白I（CTNI）1.11 ng/ml，ALT 749 U/L，AST 328 U/L，Cr 122 µmol/L，Na 159.9 mmol/L，活化部分凝血活酶时间（APTT）延长至测不出，心脏、肝脏、肾脏等多脏器受损表现。积极予抗感染、保护肝脏、扩张冠状动脉、利尿、排钠、输注冻干人纤维蛋白原、新鲜冰冻血浆等对症治疗，同时完善HLH相关检查：甘油三酯1.56 mmol/L，铁蛋白逐渐升至12 532.0 ng/ml，NK细胞活性13.88％（正常参考值≥15.11％），sCD25 930 pg/ml，FBG 494 mg/L。复查细胞因子水平：IL-1β<0.4 pg/ml，IL-6<5.8 pg/ml，IL-8 3.2 pg/ml，IL-10 1.8 pg/ml，TNF-α<5.3 pg/ml。骨髓穿刺：浆细胞偶见，吞噬细胞可见噬血现象。考虑为MAS样病变，IL-6抗体效果不佳，遂应用依托泊苷联合地塞米松控制炎症反应。予依托泊苷每周1次×4周，每2周1次×2周静脉滴注，地塞米松10 mg每日1次控制炎症，同时予丙种球蛋白、冻干人纤维蛋白原、新鲜冰冻血浆、积极抗感染及升白细胞等对症治疗后，心脏、肝脏、肾脏、凝血功能、电解质等各项指标逐渐恢复至正常，铁蛋白逐渐降至1235.3 ng/ml，患者神志逐渐好转，复查鞍区增强MRI，复测细胞因子水平：IL-1β<0.9 pg/ml，IL-6<8 pg/ml，IL-8 2.1 pg/ml，IL-10 2 pg/ml，TNF-α<4.6 pg/ml。随访过程中患者出现双耳听力较前下降，肌力从Ⅳ级恢复至Ⅴ-级，肌张力轻度减低等体征，但未再发生上述症状。

例3，女，52岁，2019年8月明确诊断为MM，IgG κ型，DS分期ⅢA期，ISS分期Ⅲ期。2019年8月开始接受VRD（硼替佐米+来那度胺+地塞米松）方案化疗，4个疗程化疗后达到VGPR，既往化疗过程顺利。2020年2月因腰背痛加重、截瘫入院。胸椎MRI：胸椎多发异常信号，肿瘤椎旁和椎管内侵犯，T7节段椎管狭窄脊髓受压。血M蛋白50 g/L，考虑疾病进展，髓外浆细胞瘤压迫脊髓致截瘫。2020年2月接受Dara-VTD（达雷妥尤单抗+硼替佐米+沙利度胺+地塞米松）方案化疗，疾病未缓解。2020年3月D-BCDDT（达雷妥尤单抗+硼替佐米+沙利度胺+地塞米松+脂质体阿霉素+环磷酰胺）方案化疗。4 d后患者出现发热，体温持续升高至38.3 °C，血钠进行性减低至122.3 mmol/L，伴腹泻、双侧胸腔积液。予托伐普坦、食盐胶囊（约15 g/d）口服联合静脉补钠治疗，以及多种抗细菌、真菌、病毒、不典型病原体等抗感染治疗，体温仍波动在38.0～39.3 °C，低钠血症可部分纠正（130～134.7 mmol/L）。2020年3月铁蛋白980.6 ng/ml；细胞因子检测：IL-1β 3.1 pg/ml（正常参考值<2 pg/ml），IL-6 24 pg/ml（正常参考值<8 pg/ml），IL-8 4 pg/ml（正常参考值<20 pg/ml），IL-10 7.2 pg/ml（正常参考值<2 pg/ml），TNF-α 18.3 pg/ml（正常参考值<8 pg/ml）；血常规：WBC 1.92×10^9^/L，HGB 69 g/L，PLT 27×10^9^/L；甘油三酯（TG）2.19 mmol/L；FBG 301.9 mg/L。结合以上症状体征及实验室检查结果，考虑可能为化疗药物引起MAS样病变及SIADH，但患者拒绝行HLH其他相关检查。因患者细胞因子轻中度升高，MAS样症状不严重，予甲泼尼龙60 mg静脉滴注治疗。患者体温波动于37.6～38.0 °C，胸腔积液减少。2020年3月复查胸椎MRI：肿瘤显著缩小，但患者双下肢肌力、感觉仍无改善，患者拒绝继续治疗要求自动出院，2020年5月于院外死亡。

## 讨论

硼替佐米在初治及复发/难治MM患者中广泛应用[2]。硼替佐米应用过程中可出现周围神经病变、腹泻、乏力、血小板减少和低钠血症等不良反应。文献报道硼替佐米用药期间低钠血症的发生率为15％～40％[1]，其具体原因尚不清楚。SIADH的存在不仅与死亡率增加有关，还使疾病的治疗复杂化[3]。

既往研究表明，IL-1β、IL-6、TNF-α等促炎性细胞因子参与炎症相关低钠血症的发生，且这一过程与抗利尿激素（ADH）的分泌有关[4]。Kim等[5]提出，NLRP3炎症小体是细胞渗透压的主要感受器，血钠降低、细胞肿胀，细胞内渗透压降低诱导炎症小体NLRP3的激活。活化的NLRP3可通过IL-1β和IL-6刺激垂体后叶释放ADH，加重低钠血症。不断降低的血钠和渗透压又持续地激活炎症小体，从而使NLRP3的活化和低钠血症成为恶性循环。Eisenhut等[6]提出炎症介质如IL-6和TNF-α通过减少肾小球基底膜上皮钠通道和（或）钠钾ATP酶的表达和抑制其功能减少上皮细胞钠的转运，并导致低钠血症。大量临床研究也证实低钠血症与多种炎症性疾病相关。Patwari等[7]报道的60例细菌性脑膜炎患者中有22例（36.7％）在入院时被诊断为SIADH，且SIADH与脑膜炎症的严重程度显著相关。Park等[8]和Il Shin等[9]首先证明了低钠血症与儿童系统性红斑狼疮（SLE）中C3降低、红细胞沉降率升高与SLE疾病活动指数（SLEDAI）存在相关性。存在低钠血症的SLE患者与无低钠血症者相比SLEDAI评分明显升高（*P*＝0.026），且在一个独立的成人SLE队列中证实了血清钠水平与血清IL-6水平呈负相关（*r*＝−0.317，*P*＝0.003）。我们报道的2例患者病程中均有不同程度的细胞因子TNFα、IL-6、IL-1β升高。抗炎治疗后患者病情改善，炎症因子水平下降，与文献报道一致。因此，我们推测IL-1β、IL-6、TNF-α等促炎性细胞因子参与了硼替佐米诱发的低钠血症，低钠血症可能与炎症因子风暴的严重程度相关。

本中心例2应用IL-6单抗拮抗炎症因子风暴后，虽然细胞因子水平显著降低，但患者铁蛋白显著升高并出现多系统功能异常，联合应用依托泊苷和地塞米松才最终控制病情。表明炎症因子风暴发生时多个细胞因子参与了对器官功能的损伤，IL-6只是其中重要的炎症因子之一。当出现多器官功能损伤时体内炎症因子风暴剧烈，单抗类药物很难遏制疾病进展，需尽快应用细胞毒药物如依托泊苷减少多种细胞因子的释放。Brito-Zerón等[10]提出，以免疫抑制剂、静脉注射免疫球蛋白、生物制剂和依托泊苷为基础治疗的HLH患者预后最好，以糖皮质激素单药治疗的患者的预后最差。此外，支持治疗也非常重要。

本中心病例显示，应用含硼替佐米方案化疗后出现低钠血症的时间不确定，临床医师应密切监测电解质的变化并及时处置。我国《老年患者低钠血症的诊治中国专家建议》[11]和《中国心力衰竭诊断和治疗指南2018》[12]均提出，对于SIADH，如果限水不能有效提高血钠水平，可选用精氨酸加压素受体拮抗剂治疗。Salahudeen等[13]的随机、双盲对照研究纳入30例癌症伴低钠血症患者，发现托伐普坦治疗组血钠纠正比例明显高于安慰剂组（94％对8％，*P*<0.001），且两组不良事件发生率和45 d内死亡率的差异无统计学意义（*P*＝0.570），充分证实了托伐普坦在治疗SIADH方面的有效性和安全性。本中心3例患者应用托伐普坦治疗可部分纠正血钠，表明硼替佐米导致的SIADH也可应用托伐普坦进行治疗。例2在应用IL-6单抗后，托伐普坦可逐渐减量至停药，血清钠恢复至正常水平，表明IL-6单抗对低钠血症有较好的疗效，间接证明IL-6可能参与了血钠调控。

含硼替佐米方案治疗MM可发生MAS样病变及严重低钠血症，患者用药过程中需密切监测血钠及体温变化。发生上述并发症时，轻症患者可首选糖皮质激素和托伐普坦治疗。如果激素应用1周后体温仍控制不佳或出现血流动力学不稳定及多脏器功能损害时，可尝试应用治疗HLH的方案联合依托泊苷及地塞米松进行治疗。因病例数较少，本病具体的发病机制及治疗方法还需要进一步研究证实。
